# Exploitation of Common Bean Flours with Low Antinutrient Content for Making Nutritionally Enhanced Biscuits

**DOI:** 10.3389/fpls.2016.00928

**Published:** 2016-06-27

**Authors:** Francesca Sparvoli, Monica Laureati, Roberto Pilu, Ella Pagliarini, Ivan Toschi, Gianluca Giuberti, Paola Fortunati, Maria G. Daminati, Eleonora Cominelli, Roberto Bollini

**Affiliations:** ^1^CNR, Institute of Agricultural Biology and BiotechnologyMilan, Italy; ^2^Department of Food, Environmental and Nutritional Sciences, University of MilanMilan, Italy; ^3^Department of Agricultural and Environmental Sciences - Production, Landscape, Agroenergy, University of MilanMilan, Italy; ^4^Alimentari e Ambientali, Facoltà di Scienze Agrarie, Istituto di Scienze degli Alimenti e della Nutrizione, Università Cattolica del Sacro CuorePiacenza, Italy

**Keywords:** amino acid score, α-amylase inhibitor, biscuits, consumers test, lectins, nutritional enhancement, phytic acid, predicted glycemic index

## Abstract

Consumption of legumes is associated with a number of physiological and health benefits. Legume proteins complement very well those of cereals and are often used to produce gluten-free products. However, legume seeds often contain antinutritional compounds, such as phytate, galactooligosaccharides, phenolic compounds, lectins, enzyme inhibitors, whose presence could affect their nutritional value. Screening natural and induced biodiversity for useful traits, followed by breeding, is a way to remove undesirable components. We used the common bean cv. Lady Joy and the *lpa1* mutant line, having different seed composition for absence/presence of lectins,α-amylase inhibitor, (α-AI) and phytic acid, to verify the advantage of their use to make biscuits with improved nutritional properties. We showed that use of unprocessed flour from normal beans (Taylor's Horticulture and Billò) must be avoided, since lectin activity is still present after baking, and demonstrated the advantage of using the cv. Lady Joy, lacking active lectins and having active α-AI. To assess the contribution of bean flour to biscuit quality traits, different formulations of composite flours (B12, B14, B22, B24, B29) were used in combinations with wheat (B14), maize (gluten-free B22 and B29), or with both (B12 and B24). These biscuits were nutritionally better than the control, having a better amino acid score, higher fiber amount, lower predicted glycemic index (pGI) and starch content. Replacement of cv. Lady Joy bean flour with that of *lpa1*, having a 90% reduction of phytic acid and devoid of α-AI, contributed to about a 50% reduction of phytic acid content. We also showed that baking did not fully inactivate α-AI, further contributing to lowering the pGI of the biscuits. Finally, data from a blind taste test using consumers indicated that the B14 biscuit was accepted by consumers and comparable in terms of liking to the control biscuit, although the acceptability of these products decreased with the increase of bean content. The B22 gluten-free biscuits, although received liking scores that were just above the middle point of the hedonic scale, might represent a good compromise between health benefits (absence of gluten and lower pGI), expectations of celiac consumers and likeness.

## Introduction

Legumes are well-recognized functional foods and their use as ingredients for food formulations is getting increasing attention (Vaz Patto et al., 2015). A number of studies has provided indications that consumption of legumes is associated with several physiological and health benefits, such as prevention of cardiovascular disease, obesity, diabetes mellitus, and cancer. These health benefits are attributed to high content in legume seeds of important soluble and insoluble fiber, to their slowly digestible starch properties as well as to prebiotic oligosaccharides, phenolic compounds and some proteins such as the lupin γ-conglutinin, and the soybean 7S globulin α**'** chain (Duranti, 2006; Sparvoli et al., 2015). Some of these components regulate glycaemia and gastrointestinal function, whereas others provide antioxidant properties (Cardador-Martinez et al., 2002). In particular, due to their poor digestibility related to the inherent physical and structural properties of starch, legumes possess lower glycemic index (GI) when compared to cereal grains, a characteristic providing benefits for people with diabetes and/or cardiovascular disease (Hoover and Zhou, 2003; Sandhu and Lim, 2008). Moreover, being gluten-free, legumes could be used as ingredients to biofortify preparations for the celiac population.

Common bean (*Phaseolus vulgaris* L.) has significant cultural and historical importance as a staple food and is essential to human diets in many parts of the world. Bean seeds are a good source of energy, complex carbohydrates (dietary fibers, starch, and oligosaccharides), proteins, important minerals and vitamins (such as iron, zinc, B-vitamins, folates) as well as antioxidants and polyphenols required for human health. From a nutritional standpoint, bean seeds are higher in proteins than cereal grains (18–24 vs. 8–15%) and the amino acid profile of seed (storage) proteins well complements that of cereals, which are normally rich in sulfur amino acids and poor in lysine, tryptophan and threonine. However, seeds of common bean also contain a number of bioactive and/or antinutritional compounds, such as lectins, digestive enzyme inhibitors, phytate, galactooligosaccharides, phenolic compounds, whose presence could affect seed nutritional value (Sparvoli et al., 2015).

In bean seeds, major lectins are the erythroagglutinating and leucoagglutinating phytohemagglutinins (PHA-E and PHA-L, respectively). These PHAs, together with the evolutionary related α-amylase inhibitor (α-AI), belong to the APA gene family of storage proteins and are inherited as a single Mendelian locus (Lioi et al., 2003). Bean α-AI, also known as phaseolamin, is able to inhibit mammalian α-amylases, thus inhibiting starch digestion by blocking access to a basic active site of the α-amylase enzyme (Santimone et al., 2004). As α-AI prevents the digestion of complex carbohydrates, it is widely used as basic active ingredient of commercial starch blocker preparations for the control of body weight (Barrett and Udani, 2011). However, α-AI has also been shown to be effective in reducing post-prandial plasma levels of glucose, insulin, C-peptide and gastric inhibitory polypeptide in healthy subjects as well as in individuals affected by diabetes mellitus (Layer et al., 1985, 1986).

Phytic acid (*myo*-inositol-1,2,3,4,5,6-hexa*kis*phosphate) is highly negatively charged at physiological pH and easily precipitates in the form of phytate salts, binding important mineral cations such as iron, zinc, potassium, calcium, and magnesium. Monogastric animals, including humans, lack phytases in their digestive tract and fail to process the phytates present in seeds, thus phytic acid is poorly digested and decreases the nutritional value of the seeds (and derived foods) by limiting phosphorus and mineral bioavailability (Schlemmer et al., 2009).

The growing body of research on legumes health benefits has stimulated interest in increasing consumption of grain legumes and thus to expand their use in food products. In particular, biscuits are the largest category of snack items among baked food worldwide and they are one of the best ways to reach all segments of the population, due to their low manufacturing cost, convenience and long shelf life. For these reasons, biscuits represent a good way to propose composite flours (Chavan and Kadam, 1993).

Common beans have been regarded as useful ingredients for the production of snacks or baked products, like biscuits, bread or pasta, especially for the development of gluten-free products (Szafranski et al., 2005; Anton et al., 2009; Siddiq et al., 2013; Manonmani et al., 2014; Giuberti et al., 2015, 2016). Incorporation of common bean flour causes a positive impact on the levels of proteins, dietary fibers, resistant starch and predicted glycemic index, pGI (Anton et al., 2009; Giuberti et al., 2015, 2016). However, their use is limited by the presence of antinutritional factors. Consumption of raw or inadequately cooked beans is well known to cause poisoning, which clinical symptoms are extreme nausea, vomiting, diarrhea, severe acute gastroenteritis and intestinal malabsorption (Noah et al., 1980; Rodhouse et al., 1990, Petry et al., personal communication). The toxicity has been ascribed to the presence of active lectins (Kumar et al., 2013), that accumulate in substantial amounts in bean seeds. Thus, the use of bean flour needs typical processing involving cooking and grinding, or extrusion and grinding, both of which introduce a heating step that would inactivate enzyme inhibitors and lectins (Elkowicz and Sosulski, 1982; Alonso et al., 2000). Inactivation and/or removal of undesirable components are essential for improving bean overall nutritional quality and acceptability, aiming to favor their potentiality as human food and/or food ingredient.

**Figure 1:**
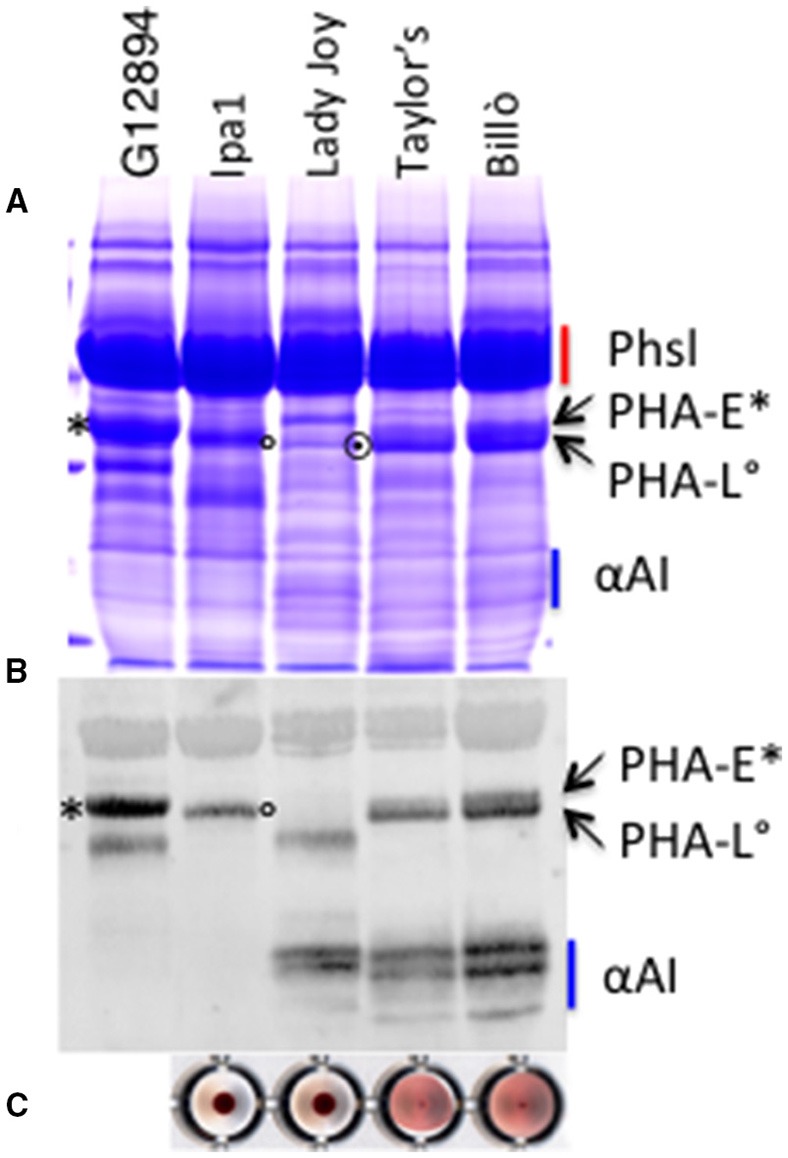
**SDS-PAGE analysis (A), immunoblot profiles (B), and hemagglutination test (C) of the seed extracts from the different genotypes used to prepare the bean containing biscuits**. G12894, wild accession containing PHA-E. The major storage proteins are indicated. Red bar, Phsl, phaseolin polypeptides; blu bar, α-AI; asterisk, PHA-E; circle, PHA-L; dotted circle, pinto lectin.

Screening of natural and induced biodiversity for useful traits, followed by breeding, is a way for removal of undesirable components. Using this approach we identified genotypes showing differences in the content of PHA-E, PHA-L, α-AI and phytic acid, and we used them as parents in breeding programs to produce lines with different sets of storage proteins (Confalonieri et al., 1992; Bollini et al., 1999; Campion et al., 2009; Figure 1).

Our purpose was to verify the advantage of using these genetic materials to make composite flours for the preparation of biscuits with improved nutritional properties. We based our work on the cv. Lady Joy and the *lpa1* mutant line. The cv. Lady Joy has an active α-AI and contains only low amounts of the so-called “pinto lectin,” an almost inactive PHA whose presence in common beans has been reported with a frequency lower than 10% (Pusztai et al., 1979, 1981; Voelker et al., 1986), consequently it has not erythroagglutinating activity (Figure 1C). The *lpa1* line carries a mutation in the gene coding for the phytic acid transporter (*Pvmrp1*) and shows a reduction of seed phytic acid of about 90% (Panzeri et al., 2011). Furthermore, this line is devoid of erythroagglutinating and α-AI activities (lacks PHA-E and α-AI), accumulating only low amounts of PHA-L (Figure 1B, compare *lpa1* with G12984, a wild bean accession only containing PHA-E, and the control cv. Taylor's Horticulture). We also used two wild type genotypes: the Italian landrace Billò, typically cultivated in Piedmont region (Piergiovanni and Lioi, 2010) and the cv. Taylor's Horticulture, that is the recurrent parent of the cv. Lady Joy.

Here, we present data demonstrating the advantage of using the cv. Lady Joy, and the *lpa1* line for the nutritional improvement of the biscuits. Having very low lectin activity or phytic acid content in their seeds, these bean flours do not need specific thermal treatments of the flour to inactivate PHA.

The resulting biscuits turned out to be nutritionally better than the control biscuit, having a better amino acid score, higher amount of fibers, lower starch content, and lower pGI. We also show that baking did not fully inactivate α-AI, further contributing to lowering the pGI of the biscuits. Finally, we provide data from a consumer blind taste test using consumers showing that the best biscuit formulation (B14) had a good acceptability score, not significantly different from control biscuit.

## Materials and methods

### Plant materials

Common bean whole flours were obtained by finely grinding the seeds with a Waring Commercial Blender(http://www.waringcommercialproducts.com/catalog.php?pcID=84_95&products_id=296). Bean flours were used as such, as no processing involving cooking and grinding, or extrusion and grinding was applied prior their use. Seeds were obtained from genotypes differing for their PHA composition and/or presence or absence of phytic acid: (i) control genotypes: cv. Taylor's Horticultural (Asgrow) and the Italian landrace Billò, a typical landrace of Cuneo, Piedmont region, both containing PHA-E, PHA-L, and α-AI; (ii) cv. Lady Joy, lacking active PHA and containing α-AI (Confalonieri et al., 1992); (iii) *lpa1* mutant line, with 90% seed phytic acid reduction and lacking PHA-E and α-AI (Campion et al., 2009). Commercial unprocessed flours of soft wheat (“00” type, http://www.molinochiavazza.it/page.php?pagina=7) and maize (“fioretto” type, http://www.molinorossetto.com/it/farine-di-mais/1862-farina-fioretto-gialla.html) were purchased from a local supplier.

### Preparation of biscuits

Biscuits were prepared by PRIMO PAN bakery (Battifollo, CN, Italy), based on the recipe of a typical Italian biscuit, from Cuneo region, called “Pasta di meliga” (http://www.primopan.com/fogliemais.html). This typical biscuit is made with composite flour containing 67 and 33% of soft wheat and maize flours, respectively (Table 1, control). The ingredients for biscuits preparation included: 540 g of composite flour, 300 g raw cane sugar, 300 g butter, 2 medium eggs (ca. 110 g), 2 g common salt, 14 g baking powder, natural vanilla flavor (Table 1). Biscuits were baked at 180°C for 12 min. In addition to the control, five different types of biscuit samples were prepared (B12–B29) replacing maize and/or wheat with different amounts of common bean flours as described in Table 1.

**Table 1:** **Main ingredients composition of biscuit samples**.

**Sample**	**Flours composition**						**Total flours (g)**				**Dough weight (g)**
	**g**			**% of dough weight**				**Other ingredients (g)**			
	**Wheat**	**Maize**	**Bean**	**Wheat**	**Maize**	**Bean**		**Eggs**	**Butter**	**Sugar**	
Control	360	180	−	29	14	−	540	110	300	300	1250
B12	240	150	150	19	12	12	540	110	300	300	1250
B14	360	−	180	29	−	14	540	110	300	300	1250
B22	−	270	270	−	22	22	540	110	300	300	1250
B24	120	120	300	10	10	24	540	110	300	300	1250
B29	−	180	360	−	14	29	540	110	300	300	1250

### Hemagglutination test

Hemagglutinating activity in the extracts was estimated by a serial dilution procedure using human type A erythrocytes suspension (0.5% in phosphate buffered saline buffer pH 7.0, PBS) adding 50 μl of sample to the same volume of human erythrocytes. Agglutination was visually determined after 4 h incubation at room temperature. For each sample, serial dilutions in PBS, ranging from 1:10 to 1:1280, of seed and biscuit extracts were assayed. Results were recorded by monitoring how many 1:1 dilutions of the sample were necessary to prevent agglutination of red blood cells. Hemagglutinating unit (HAU) is defined as the reciprocal of the highest dilution still showing by eye agglutination of red cells, according to Trugo and von Baer (1998).

### Protein extraction, SDS-PAGE and immunoblot analyses

Total seed proteins extraction from dry bean seeds and biscuits, using 10 volumes of 20 mM borate pH 9, and separation by electrophoresis on an 15% sodium dodecylsulfate-polyacrylamide gel (SDS-PAGE) were as described by Bollini and Chrispeels (1978). Gels were stained with Coomassie Brilliant Blue R-250 or blotted on a supported nitrocellulose membrane (Hybond-C, GE-Healthcare). Immunoblot analysis was performed according to Burnette (1981) using rabbit antibodies against *P. vulgaris* recombinant α-AI at 1:1000 dilution. These antibodies allow the detection of both PHA and α-AI (Ceriotti et al., 1989). Peroxidase-linked anti-rabbit IgG was used as the secondary antibody.

### Assay of α-amylase inhibitor activity

Inhibitory activity against human salivary α-amylase (EC 3.2.1.1; Type IX-A) was measured by the increase of iodine staining after the action of the α-amylase enzyme on soluble starch as described in Altabella and Chrispeels (1990). Briefly, different volumes (from 10 to 100 μl) of biscuit flour or seed extracts diluted 50 or 200 fold in 20 mM borate buffer pH 9, respectively, were preincubated with a fixed amount of α-amylase (0.15 U) for 30 min at room temperature in a final volume reaction of 300 μl. Then, 200 μl of a 0.15% solution of potato starch was added and after 5 min at room temperature the reaction was stopped by adding 1 ml of Iodine reagent (Varner and Mense, 1972) and absorbance was measured at 620 nm. Results were expressed as units of α-amylase inhibited per mg of flour, where one unit of inhibitor activity is the amount which will bring about 50% inhibition of the α-amylase in 30 min under the above conditions according to Marshall and Lauda (1975).

### Determination of phytic acid phosphate

Phytic acid phosphate (PAP) fraction was determined by a ferric precipitation method (Pilu et al., 2003) and expressed as μg/mg of sample. The amount of corresponding phytate level was then obtained by multiplying these values for the conversion factor 3.5484.

### Bromatological analysis and *in vitro* starch digestion of experimental biscuits

The chemical composition of biscuit samples was assayed according to AOAC standard methods (AOAC, 2000). Samples were milled and analyzed for dry matter (DM), ash, crude protein (CP), and ether extract (EE); starch content was measured enzymatically. Neutral detergent fiber (NDF), acid detergent fiber (ADF), and acid detergent lignin (ADL) were determined using the filter bag system (ANKOM Technology, New York, USA). In particular, NDF was assessed according to the procedure of Mertens (Mertens et al., 2002), while ADF and ADL were determined according to Vansoest et al. (1991). Gross energy was measured using an adiabatic calorimeter (IKA 4000, Staufen, Germany). Total amino acid composition was evaluated on defatted flours according to Commission Regulation (EC) No 152/2009 of 27 January 2009 laying down the methods of sampling and analysis for the official control of feed.

The multi-enzymatic protocol detailed by Giuberti et al. (2015) was used to evaluate the *in vitro* starch digestion of biscuits over time. The method, based on a 2-step enzymatic digestion incorporating a gastric (pH = 2) and a pancreatic (pH = 5.2) phase, has been shown to predict GI values with good accuracy for several cereal- and legume-based foods. Briefly, milled samples (800 mg) were carefully inserted into 50 ml tubes containing glass balls and pre-treated with a 0.05 M HCl solution (5 ml) containing pepsin (5 mg/ml; Sigma P-7000, Sigma Aldrich Co., Milan, Italy) for 30 min at 37°C under agitation. After the simulation of the gastric digestion, the pH of the solution was adjusted to 5.2 by adding 20 ml of 0.1 M acetate buffer. Then, 5 ml of an enzyme mixture (amylase activity of about 7000 U/ml; Giuberti et al., 2015) given by pancreatin (Merck 7130; Merck KGaA, Darmstadt, Germany), amyloglucosidase (Sigma A-7095; Sigma Aldrich Co., Milan, Italy) and invertase (Sigma I-4504; Sigma Aldrich Co., Milan, Italy) enzymes was added. From each tube, aliquots were carefully taken at 0 (prior to the enzyme addition simulating the pancreatic phase) and at 30, 60, 120, and 180 min after the enzyme addition. To each collected aliquot, absolute ethanol was added in order to stop the enzyme hydrolysis and the amount of released glucose was determined colorimetrically (glucose oxidase kit GODPOD 4058; Giesse Diagnostic snc, Rome, Italy). Commercial fresh white bread (starch content of 72.3% DM) was used as reference and a blank was also included to correct for the glucose in the amyloglucosidase solution. The percentage of digested starch at each time interval was calculated using a factor of 0.9 to convert mono to polysaccharides. For each treatment, samples were analyzed in duplicate. After the enzyme digestion, a hydrolysis index (HI) was derived from the ratio between the area under the hydrolysis curve (AUHC) of each biscuit samples and the corresponding AUHC of the reference fresh white bread as a percentage over the same period. From the obtained HI, a predicted GI value was then calculated with the formula pGI = 8.198 + 0.862 × HI (Granfeldt, 1994).

### Consumer test

The acceptability of the six different types of biscuits was assessed involving 102 subjects (55 females and 47 males, age: mean = 24.0; sd = 5.3). Consumers were recruited according to their liking for and regular consumption of biscuits. These subjects were asked to taste one biscuit of each sample, and to express their liking according to a 100-mm linear hedonic scale anchored at the extremes with “dislike extremely” (left of the scale, score = 0) and “like extremely” (right of the scale, score = 100). In order to balance the effects of serving order and carry-over, samples presentation order was systematically varied over participants (Macfie et al., 1989). Samples were served at room temperature (about 20°C) in plastic plates coded with 3-digit numbers and evaluated in individual booths in white light conditions. Participants were asked not to smoke, eat or drink anything, except water, for 1 h before the tasting sessions.

The study complied with the principles established by the Declaration of Helsinki and the protocol was approved by the Ethics Committee of the University of Milan. Written informed consent was obtained from each subject before the liking assessment was performed.

## Results

### Evaluation of erythroagglutinating activity in bean based biscuits

Usually, bean seeds accumulate substantial amounts of PHA (both L and E types) and α-AI. Since consumption of raw or inadequately cooked beans may cause poisoning, to verify the role of baking on PHA activity we used different type of bean flours with or without PHAs. We compared different bean genotypes: (i) cv. Lady Joy flour, devoid of active lectin, (ii) its parental line, cv. Taylor's Horticulture, containing both PHA-E and PHA-L, (iii) another commercial landrace, Billò, also containing both PHA-E and PHA-L, (iv) the *lpa1* genotype, containing only PHA-L (not erythroagglutinating).

**Figure 2:**
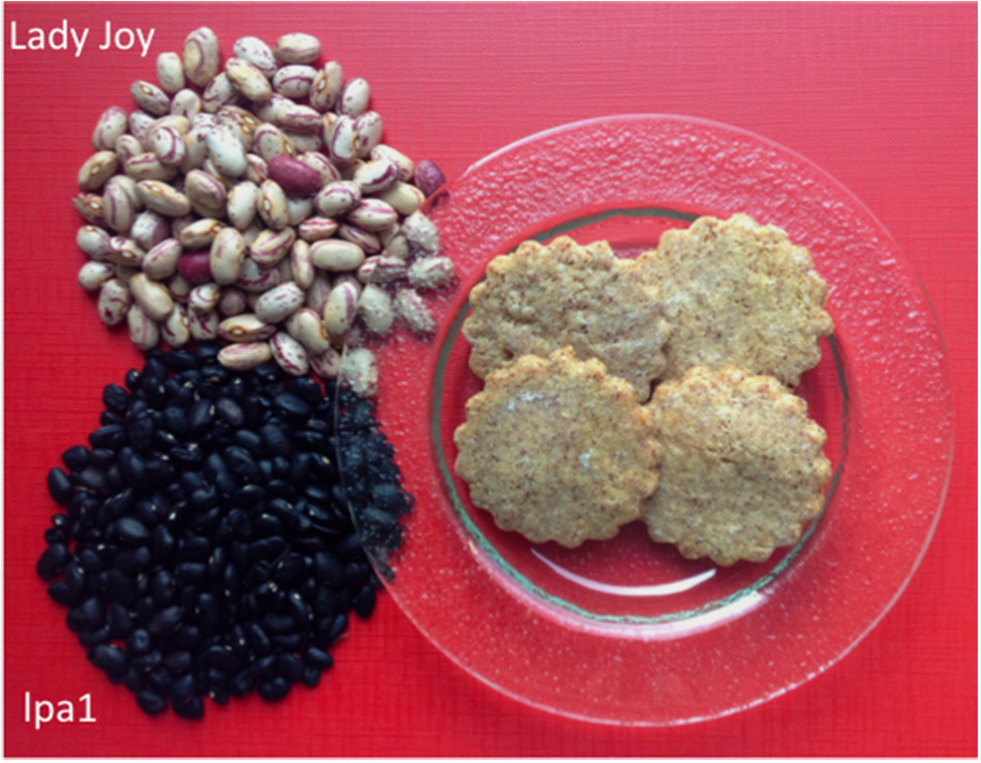
**Appearance of bean biscuits and bean seeds of the cv. Lady Joy and *lpa1* line**.

Biscuits were prepared according to the recipe of a typical Italian biscuit, from Cuneo region, called “Pasta di meliga,” in which we replaced maize with bean (B14 formulation, Figure 2, Table 1).

**Figure 3:**
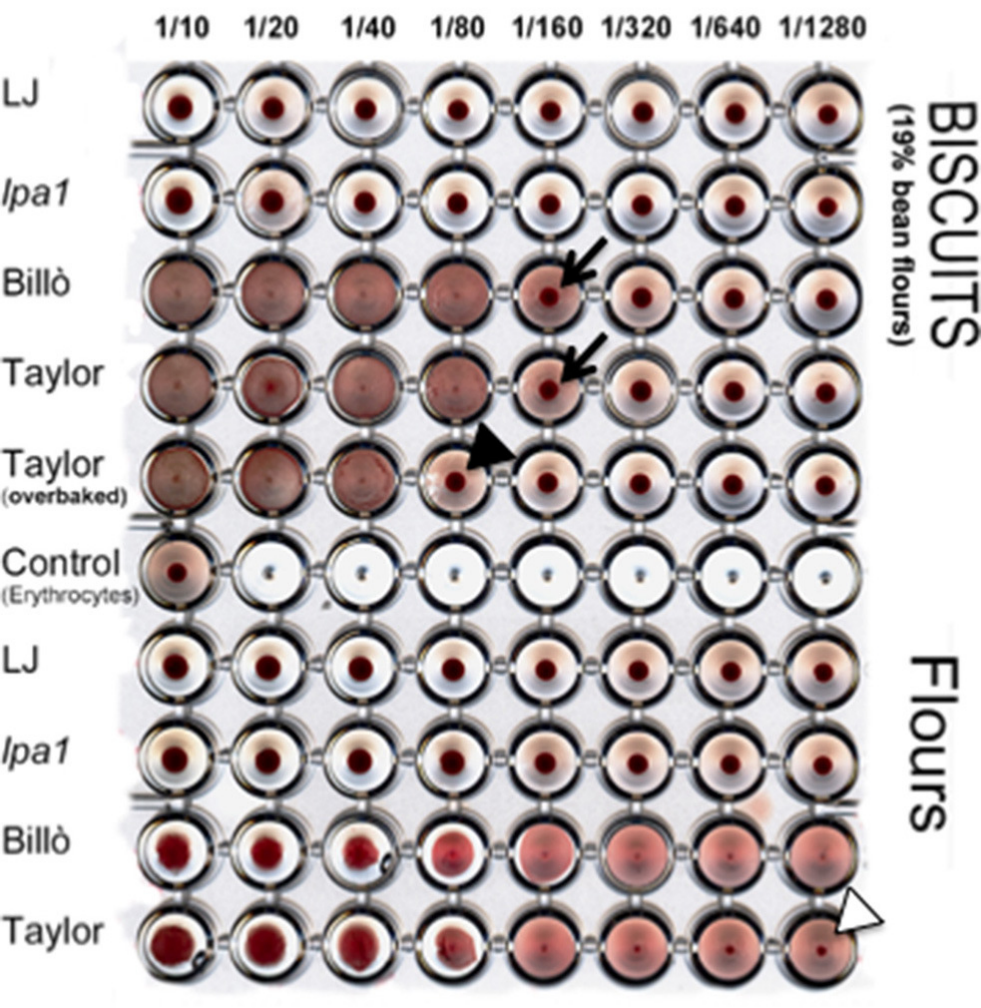
**Hemagglutination test of extracts from defatted flours of sample biscuits (B14 type) and corresponding bean seed flours of cv. Lady Joy (LJ), *lpa1* mutant line, Billò landrace and cv. Taylor's Horticulture**. Control shows erythrocytes incubated with an equivalent voulme of PBS buffer, instead of the seed extract. Arrows and arrowheads indicate the agglutination limit. Samples dilutions are indicated.

The presence of active PHA was assessed using the erythroagglutination test (Figure 3). Results show that after baking, extracts of biscuits containing PHA (cv. Taylor's Horticulture and Billò) are still able to agglutinate erythrocytes after a serial dilution between 1:80 and 1:160 (Figure 3, black arrows). Three minutes of overbaking (baking from 12 to 15 min) decreased the erythroagglutinating activity to 1:40 dilution (Figure 3, black arrowhead), confirming that proper heat treatment is needed for inactivation. As expected, no agglutination was detected on extracts of biscuits containing cv. Lady Joy or *lpa1* flours.

To quantify the residual PHA activity we compared the agglutination results with those made on corresponding amounts of unprocessed bean flours. We calculated that defatted extracts of B14 biscuits contain about 19% of bean flour. Since at least three more 1:1 dilutions are needed to abolish agglutination in equivalent cv. Taylor's Horticulture and Billò extracts (Figure 3, white arrowhead), we estimated that the residual PHA activity in these biscuits was in the range of 5–10%.

### Bean biscuits formulation

To assess the contribution of bean flour to biscuits quality traits, different formulations of composite flours were used in which wheat and/or maize flours were replaced by bean flour in the recipe of the biscuit “Pasta di meliga” (Table 1). To avoid any risk of poisoning due to residual active PHA after baking, we used the flour of cv. Lady Joy, which contains low amounts of the non-toxic “pinto lectin” and has an active α-AI. Since celiac disease has been associated with high incidences of diabetes mellitus (Lamacchia et al., 2014) and there is considerable interest in lowering the GI of gluten free foods for the maintenance of a good glycemic control, two types of biscuits, in which bean and maize flours completely replaced that of wheat (B22 and B29, Table 1), were also prepared.

Composite flours contained different percentages of bean flour: 26.7, 32.1, 50.0, 53.6, and 64.3% (B12, B14, B22, B24, B29, respectively), in combinations with wheat (B14), maize (B22 and B29), or with both wheat and maize flours (B12 and B24). Other ingredients included eggs, butter and sugar cane (Table 1). Biscuits were baked for 12 min at 180°C.

**Figure 4:**
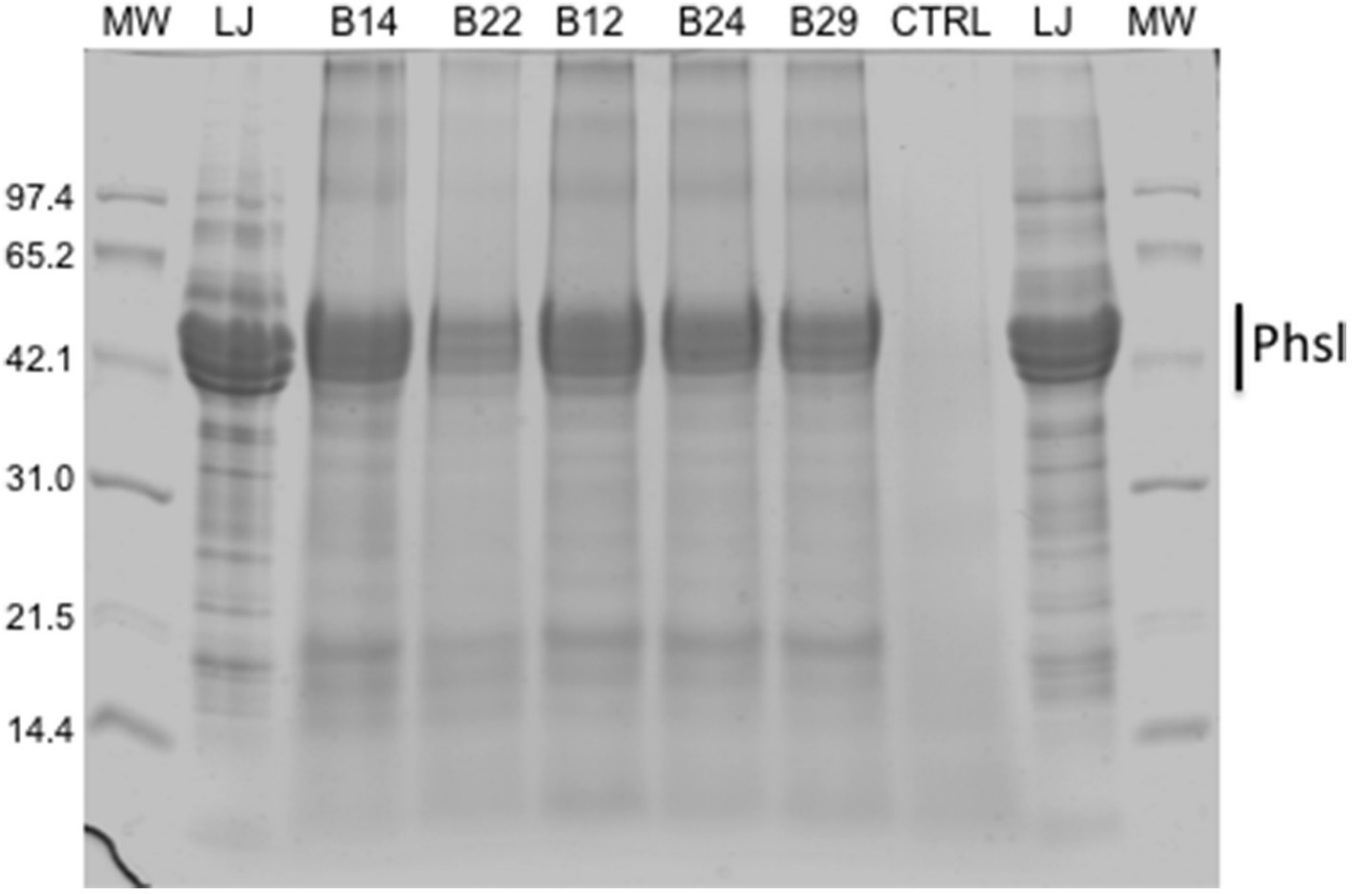
**SDS-PAGE analysis of extracts from defatted flours of sample biscuits (B14-B29, CTRL, control biscuit) and cv. Lady Joy seed extract (LJ). MW molecular weight markers**. Bar indicates phaseolin polypeptides (Phsl).

In order to assess the effect of baking on bean proteins, biscuit protein extracts were analyzed by SDS-PAGE (Figure 4). Results showed that in all the formulations the protein profile was similar to that of cv. Lad Joy seed extract, with phaseolin being the most abundant protein, as in common bean flours (Figure 4, compare LJ with B12, B14, B22, B24, B29). This result indicates that in the biscuits bean proteins have undergone poor hydrolysis.

### Analysis of α-amylase inhibitor activity and phytic acid content in biscuits

To verify the role of baking also on α-AI, its activity was analyzed in defatted extracts of B12, B14, B22, B24, B29 and control (“Pasta di meliga”) biscuits as well as in B14 biscuits made with flours of the cv. Taylor's Horticultural (Lady Joy recurrent parent) and the landrace Billò.

No relevant activity was present in control biscuits (not containing bean flour), while consistent α-AI activity was measured in extracts of all biscuit types containing bean flours (Table 2). We observed that the level of α-AI activity did not correlate with the amount of bean flour in the biscuits. In fact, the highest α-AI activity was recorded in the B22 biscuit, which is not the one with the highest bean content. We then compared α-AI activities of the different biscuits with those present in the corresponding raw bean flours. Data showed that samples B12, B24, and B29 retained a α-AI activity between 49.4 and 62.5%, while higher activities were detected in samples B14 and B22, having 100 and 75.9% of the expected α-AI activities, respectively (Table 2).

**Table 2:** **α-amylase inhibitory activity in extracts of defatted biscuit flours and bean flours of cv. Lady Joy (LJ), cv. Tayolor's Horticultural (TH), Billò (B), the *lpa1* mutant line, and over baked (OB)**.

	**Sample**	**% bean flour in defatted biscuit**	**U α-AI/100 mg bean flour**	Expected U α-AI/100 mg defatted biscuit flour^*^	**Measured U α-AI/100 mg defatted biscuit flour**	**% of expected U α-AI**
Flour	cv. Lady Joy	–	1048.42 ± 94.5			
Biscuits	Control	0		0.00	37.56± 41.28	0.00
	B12	16		167.75	94.53± 20.44	56.35
	B14	19		199.20	203.98± 8.060	102.40
	B22	28		293.56	222.73± 40.77	75.87
	B24	32		335.50	209.61± 18.67	62.48
	B29	38		398.40	196.64± 37.90	49.36
Flours	cv. Lady Joy		1132.47 ± 161.7			
	cv. Taylor's H.		1226.04 ± 164.0			
	Billò		1181.99 ± 76.9			
	*lpa1*		0.00			
Biscuits	B14 (LJ)	19		215.17	234.96± 23.50	109.20
	B14 (TH)	19		232.95	160.68± 60.06	68.98
	B14-OB (TH)	19		232.95	48.65± 2.4	20.88
	B14 (B)	19		224.58	169.16± 48.87	75.32
	B14 (*lpa1*)	19		0.00	0.00	0.00

Units of inhibited α-amylase are per 100mg of flours (U α-AI/100 mg).

* Values are the product of α-AI activity in bean flour x the percent of bean flour in defatted biscuit. B12-B29: see Table 1.

We assayed α-AI activity also on extracts of B14 biscuits made with cv. Taylor's Horticulture and Billò flours. These biscuits retained about 34% of the starting α-AI activity, while overbaking reduced α-AI activity to about 10%. As a control, we measured the α-AI activity in the cv. Lady Joy B14 sample and confirmed that 100% of α-AI activity was present (Table 2).

Phytic acid is a well-known antinutrient and is very stable to heat treatment. The use of flours almost devoid of phytic acid could therefore significantly improve the nutritional quality of baked products. To evaluate this point we quantified phytic acid in extracts of B14 biscuits made with flour of the *lpa1* mutant, which is almost devoid of phytic acid, or with those of cv. Lady Joy, cv. Taylor's Horticulture, having normal phytic acid content (Table 3).

**Table 3:** **Phytic acid (PA) content in biscuit samples (defatted flours) and bean flours of the *lpa1* mutant line, cv. Lady Joy and cv. Tayolor's Horticultural**.

	**Sample**	**PA mg/g flour**
Biscuits	B14 (*lpa1*)	1.91± 0.03
	B14 (LJ)	4.63± 0.81
	B14 (Taylor's H.)	3.78± 0.65
Flours	*lpa1*	2.38± 0.05
	Lady Joy cv.	9.32± 0.23
	Taylor's H. cv.	10.53± 1.01

Results showed that biscuits made using *lpa1* flour contained less phytic acid (1.91 mg/g) than biscuits made with flours of the other two genotypes (4.63 and 3.78 mg/g). The reduction is 58.7% compared to phytic acid content of biscuits made with cv. Lady Joy, and 49.5% if compared to biscuits made with cv. Taylor's Horticulture (Table 3).

### Proximate composition, *in vitro* predicted glycemic index classification and amino acid composition

The different biscuits were also analyzed for their proximate composition, pGI, and total amino acid content (Tables 4, 5, respectively). Compared to control biscuits, the addition of bean flour increased protein content, from a minimum of 14% (B12) up to 45.7% (B29), as well as ashes, neutral detergent fiber (NDF), acid detergent fiber (ADF) and, to a lower extent, lignin and crude fiber. The addition of bean flour to the recipe also affected starch content, which decreased from a minimum of 25.8 (B12) to 37.0% (B29) (Table 4). Lastly, pGI values (calculated using commercial white wheat bread as reference; pGI = 94.4) decreased with the addition of bean flour in the composite, ranging from 88.9 to 61.9 for control and B29 biscuits, respectively.

**Table 4:** **Proximate composition and *in vitro* predicted glycemic index of biscuit samples (g/100 g) [1 biscuit is about 11.6 ± 0.4 g; Lady Joy cv. (LJ), *lpa1* mutant line (*lpa1*), Tayolor's Horticultural cv. (TH), Billò (B)]**.

**Sample**	**Dry matter**	**Ashes**	**Crude proteins**	**Ether extract**	**NDF^a^**	**ADF^b^**	**Lignin**	**Crude fiber**	**Total sugars**	**Starch**	**GE (kJ/g)^c^**	**pGI^d^**
Control	97.1 ± 0.03	1.21 ± 0.03	7.07 ± 0.00	22.4 ± 0.26	1.40 ± 0.06	0.96 ± 0.07	0.77 ± 0.11	1.80 ± 0.13	31.2 ± 0.06	41.9 ± 0.96	22.4 ± 0.230	89.0 ± 0.35
B12	94.6 ± 0.04	1.87 ± 0.00	8.07 ± 0.23	24.4 ± 0.15	3.44 ± 0.17	2.07 ± 0.01	0.80 ± 0.00	1.70 ± 0.05	30.3 ± 0.14	31.1 ± 0.07	22.0 ± 0.005	80.6 ± 0.99
B14	95.7 ± 0.03	2.09 ± 0.00	9.24 ± 0.21	24.5 ± 0.49	2.23 ± 0.08	2.05 ± 0.03	0.77 ± 0.01	2.06 ± 0.26	30.9 ± 0.61	29.8 ± 0.02	22.6 ± 0.001	74.2 ± 1.34
B22	94.8 ± 0.00	2.21 ± 0.02	8.79 ± 0.02	24.5 ± 0.50	4.25 ± 0.68	3.04 ± 0.06	0.89 ± 0.18	1.90 ± 0.12	31.9 ± 0.76	30.5 ± 1.17	22.5 ± 0.090	70.7 ± 0.57
B24	95.3 ± 0.02	2.03 ± 0.03	8.94 ± 0.04	26.3 ± 0.10	3.45 ± 0.16	2.67 ± 0.03	0.92 ± 0.04	1.61 ± 0.23	31.1 ± 0.88	28.5 ± 0.31	22.6 ± 0.070	66.2 ± 0.92
B29	95.0 ± 0.12	2.32 ± 0.01	10.3 ± 0.26	23.3 ± 0.14	3.68 ± 0.17	2.98 ± 0.05	0.87 ± 0.22	2.28 ± 0.15	32.1 ± 0.77	26.4 ± 0.20	22.2 ± 0.180	61.9 ± 1.41
B14 (LJ)	92.9 ± 0.17	1.74 ± 0.14	11.2 ± 0.09	21.6 ± 0.09	nd	nd	nd	nd	30.2 ± 0.20	34.3 ± 0.58	nd	66.0 ± 0.71
B14 (*lpa1*)	94.4 ± 0.06	1.87 ± 0.14	12.0 ± 0.08	19.5 ± 0.58	nd	nd	nd	nd	29.5 ± 1.10	32.2 ± 0.53	nd	70.3 ± 0.21
B14 (TH)	94.5 ± 0.06	2.05 ± 0.14	11.3 ± 0.05	20.9 ± 0.33	nd	nd	nd	nd	30.3 ± 0.69	29.9 ± 0.53	nd	64.6 ± 1.70
B14 (B)	91.6 ± 0.08	1.74 ± 0.14	13.3 ± 0.02	21.6 ± 0.49	nd	nd	nd	nd	29.9 ± 0.78	29.6 ± 0.54	nd	64.5 ± 2.19

B12–B29: see Table 1.

Values are mean ± standard deviation of three independent determinations (dry weight basis), except for total sugars, the in vitro glycemic index classification, which were run in duplicate.

a NDF, Neutral Detergent Fiber (cellulose, emicellulose, lignin).

b ADF, Acid Detergent Fiber (cellulose, lignin).

c GE, Gross Energy.

d pGI, predicted Glycemic Index, calculated using commercial white wheat bread as reference (pGI = 94.4).

**Table 5:** **Total amino acid composition of biscuit samples (g/100 g defatted flour) and bean flours of cv. Lady Joy (LJ), cv. Tayolor's Horticultural (TH), and *lpa1* mutant line**.

**Sample**	**Thr**	**Lys**	**Trp**	**Cys**	**Met**	**Phe**	**Val**	**His**	**Ile**	**Asp**	**Glu**	**Pro**	**Gly**	**Ala**	**Leu**	**Tyr**	**Arg**	**NH4**	**Hexan extract % DM**	**Proteins % DM**
Control	0.337	0.144	0.041	0.175	0.141	0.379	0.496	0.229	0.323	0.572	2.354	0.940	0.299	0.395	0.722	0.300	0.309	0.268	21.4	8.75
B12	0.371	0.314	0.057	0.170	0.170	0.496	0.625	0.220	0.404	0.745	2.318	0.942	0.357	0.455	0.809	0.352	0.569	0.302	23.6	10.05
B14	0.461	0.485	0.096	0.165	0.173	0.590	0.713	0.325	0.509	0.809	2.748	1.277	0.446	0.478	0.876	0.378	0.568	0.401	22.8	12.03
B22	0.472	0.454	0.058	0.156	0.189	0.575	0.544	0.353	0.475	1.020	1.947	1.312	0.405	0.580	1.005	0.405	0.557	0.238	23.4	11.28
B24	0.473	0.491	0.080	0.168	0.185	0.591	0.757	0.352	0.488	1.025	2.251	1.430	0.438	0.551	0.939	0.401	0.599	0.316	22.5	11.91
B29	0.532	0.540	0.084	0.164	0.194	0.656	0.724	0.370	0.531	1.141	2.239	1.392	0.469	0.614	1.063	0.438	0.643	0.251	24.0	12.85
LJ	1.005	1.602	0.223	0.261	0.326	1.327	1.272	0.798	1.086	2.442	3.799	3.973	0.932	1.037	1.792	0.703	1.463	0.313	−	24.86
TH	1.186	1.741	0.243	0.252	0.307	1.526	1.416	0.819	1.241	2.985	4.300	5.037	1.043	1.120	2.024	0.722	1.793	0.374	−	28.20
*lpa1*	1.120	1.861	0.228	0.282	0.334	1.521	1.377	0.875	1.269	3.010	4.638	5.334	1.073	1.104	2.130	0.748	1.851	0.396	−	29.11

As we found that B14 biscuits retain almost 100% of the activity of the α-AI present in bean flour, we compared pGI values of B14 biscuits made with different bean flours. In order to estimate the contribution of the active α-AI to the pGI, we used as a reference the pGI of biscuits made with the *lpa1* mutant, which is devoid of α-AI (Table 2). Our results showed that B14 biscuits containing active α-AI have lower pGI values than B14 biscuits made with flour of the *lpa1* mutant, thus indicating that active α-AI slightly contributes to the decrease of the pGI value (Table 4).

The presence of bean flour in the composite flours impacted also the amino acid composition of the biscuits: the highest effect was on the content of the essential amino acids Lys, Trp, and Arg, which compared to control biscuits raised one to two fold for Lys (118–275%), 39–134% for Trp, and 84–108% for Arg. The average (B12–B29) increase for all the remaining amino acids was ranging from about 30% (Leu) up to 53% (Phe). The only slight decrease was observed for Glu, whose content decreased between 1.5 and 17.3% (B12, B22, B24, and B29) with the exception of B14 (16.7% increase), suggesting that wheat significantly contributes more than maize to the content of this amino acid (compare B14 with B29). The overall content of sulfur amino acids (Met and Cys) was not very much affected by bean flour addition and their average content increased between 7.6 and 13.3% (B12–B29) with Met contributing more than Cys (average 30% more and 6% minus, respectively; see Table A1).

### Consumer test

The introduction of a new ingredient, the bean flour, to a conventional food, such as biscuits, could result in a product that might not fully meet the acceptability of the consumers. To verify the liking of the biscuits containing increasing amounts of cv. Lady Joy bean flour, their acceptability was tested in comparison with the traditional “Pasta di meliga” (control), in a blind taste test on a consumer sample of 102 subjects. Hedonic data were subjected to ANOVA considering samples (*n* = 6) as source of variation and liking scores as dependent variable. Mean hedonic values for the six biscuits (one control and five containing different amounts of bean flour) are reported in Table 6. ANOVA results showed a significant effect of the factor samples [*F*_(5, 505)_ = 11.52, *p* < 0.001] on liking ratings. LSD *post-hoc* comparison indicated that the B14 biscuits received an acceptability score that was statistically comparable to the control biscuit, and the acceptability of B12 biscuits, although statistically different from the control, was not statistically different from that of B14 biscuits, indicating that these two types were well liked by consumers. However, liking scores decreased with the increase of the bean flour content as the B22 (gluten-free) and B24 formulations were significantly less liked and received liking scores that were just above the middle point of the hedonic scale, although they were not significantly different from the B12 sample. The B29 biscuit, gluten-free but with the highest addition of bean flour, was the least liked sample.

**Table 6:** **Results of the consumer test (*n* = 102) for evaluating the acceptability of the different bean biscuits compared to the control biscuit “Pasta di meliga”**.

**Sample**	**Mean ± SEM**	**LSD *post-hoc* groups**
Control	69.8 ± 2.3	d
B12	60.7 ± 2.5	bc
B14	64.6 ± 2.5	cd
B22	54.5 ± 2.5	b
B24	55.1 ± 2.7	b
B29	45.5 ± 2.7	a

Identical letters indicate there is no statistically significant difference between the samples according to LSD post-hoc test. Consumers liking was expressed according to a 100-mm linear hedonic scale anchored at the extremes with “dislike extremely” (left of the scale, score = 0) and “like extremely” (right of the scale, score = 100).

## Discussion

The increasing awareness of the link between diet and health is the driving force to develop novel foods to meet consumer's needs, including the identification, development and improvement of new ingredients for food formulations.

In this work we assessed the advantage of using flours of nutritionally enhanced common bean genotypes for the production of bean based biscuits, with the aim to contribute to the diversification of healthier and more nutritional diet.

### Importance of seed nutrients genetic modulation for biscuits quality and properties

A number of studies reported improved nutritive value of high protein biscuits made using composite flours, including different amounts of flour from soybean, field pea, lentil, chickpea, lupin, and common bean (Shrestha and Noomhorm, 2002; Rababah et al., 2006; Zucco et al., 2011). In general, the consumption of common bean has not gone beyond the traditional processing and uses, due to the antinutritional factors present in the seeds. Extrusion, thermal treatments, such as roasting, and soaking at high temperature, followed by cooking and dehydration, are often used to eliminate lectin and enzyme inhibitors activities (Hoojjat and Zabik, 1984; Costa et al., 2006; Boye et al., 2010; Kelkar et al., 2012; Siddiq et al., 2013). However, these types of processing are a source of additional costs for the producers and may lead to undesirable functional and sensory properties of the common bean flours (Batista et al., 2010). As a consequence, common bean is not a very common legume for flour fortification, despite its good protein and micronutrient content.

Here, we recommend the use of the cv. Lady Joy, containing an almost inactive and low abundant PHA (Pusztai et al., 1981; Confalonieri et al., 1992), to avoid any possible risk of lectin poisoning due to frequent and abundant consumption of these bean biscuits. In fact, we provide evidence that biscuits made with bean flours containing active PHA (Taylor's H. and Billò) still retain residual lectin activity (about 5–10% of the lectin in the original flours), while no agglutination is detected in biscuits made using flour of the cv. Lady Joy (Figure 4).

Another advantage of avoiding preventive thermal treatments of the bean flours (possible if using the cv. Lady Joy) was the finding that substantial levels of α-AI activity were retained in the bean biscuits, with the B14 (LJ) type showing the highest levels (Table 2). It is known that α-AI and, in general, storage proteins belonging to the *P. vulgaris* lectin family, are stable proteins resistant to thermal degradation (Sparvoli et al., 1997). Nevertheless, the finding that cv. Lady Joy biscuits B14 retained such a high α-AI activity was unexpected. A possible explanation could be that dry heating, as it occurs during baking, induces changes in the physicochemical properties of the dough and consequently of its components, causing enhancement of the α-AI activity as has been shown for microwave treatment of beans (Oomah et al., 2014). The presence of active α-AI was confirmed in cv. Taylor's Horticulture and Billò B14 biscuits, although to a lesser extent. This might be due to the fact that these two genotypes contain a different α-AI isoform that might differently respond to heat treatments (Bollini et al., 1999).

### Biofortified bean biscuits: improved protein content and amino acid score, and reduced phytic acid content

All biscuit formulas with composite bean flours showed a better nutritional composition, compared to the control, i.e., higher levels of protein and fiber, better amino acid score, and lower starch content along with a reduction in the pGI values. A similar behavior should be expected in food preparation containing different percentage of legume flour, as already reported for rice spaghetti, semolina spaghetti and teff tagliatelle prepared with increasing levels of bean flour (Gallegos-Infante et al., 2010; Giuberti et al., 2015, 2016). In addition, present findings are consistent with data obtained by other authors (Rababah et al., 2006; Zucco et al., 2011). For example, fortification with 12% of broad bean flour resulted in a protein increase from 16.6 to 20.2% (Rababah et al., 2006). Our results also indicate that the presence of maize in composite flours decreased biscuit protein value, as B14 biscuits, not containing maize, have higher protein content than B22–B29 samples (Tables 4, 5). Indeed, this is in accordance to the fact that wheat grains have average protein content higher than that of maize, traditionally an oil crop.

The nutritional enhancement of bean-containing biscuits was evident also for the amino acid composition, as the addition of bean flour increased the content of limiting essential amino acids (Lys, Thr, Trp) without lowering the content of sulfur amino acids. Furthermore, the replacement of cv. Lady Joy bean flour with that of *lpa1* contributed to about a 50% reduction of phytic acid content in B14 biscuit formulations. Nutritional studies performed on volunteers indicated that iron absorption from *lpa1* beans is significantly higher than from their parents with normal phytic acid levels (Petry et al., 2013, 2015). These results indicate that the B14 formulation containing *lpa1* flour is suited to produce biofortified biscuits with an enhanced and more balanced protein content and with increased bioavailability of essential minerals.

One third of the global population is estimated to suffer from hidden hunger. Micronutrient deficiencies vary in severity and occur in every country, but they disproportionally affect women, children, and people living in developing countries (Von Grebmer et al., 2014). Thus, malnutrition and hidden hunger represent a big nutritional challenge. The bean biscuits we presented here could be further implemented and used as a biofortified food snack, especially for children, using the *lpa1* bean flour in the B29 formulation, together with a flour from a biofortified maize *lpa1* mutant (Cerino Badone et al., 2012; Landoni et al., 2013). In this case, we expect a novel food product having a very high mineral bioavailability and higher and balanced protein content (Tables 2, 3, A1).

### Biofortified bean biscuits: predicted glycemic index and consumer's test

The classification through the pGI can be useful to predict the likely *in vivo* glycemic response of food of interest and a reference material. Accordingly, foods can be categorized into low (< 55), medium (55–69), and high GI (>70) (Foster-Powell et al., 2002). With respect to the control, the pGI values of experimental biscuits decreased with the addition of bean flour, as already reported by Giuberti et al. (2015) where the presence of 20 and 40% of bean flour in gluten-free rice pasta reduced the pGI from 61, in the control, to 37 and 31, respectively. With respect to cereal flours, starch from bean is characterized by higher level of amylose and by larger size of starch granules, both starch structural properties that can contribute to impede overall starch degradability (Sandhu and Lim, 2008). Moreover, comparative studies reported that cereal starches are characterized by the A-type power diffraction pattern, which has an open structure rendering cereal starches highly digestible (Biliaderis, 1991). On the contrary, legume starches tend to exhibit the C-type crystallinity, which is more resistant to digestion due to a close-packed structure (Biliaderis, 1991). Lastly, the increasing amounts of fiber and protein found in bean-enriched biscuits, along with the possible formation of amylose-lipid complexes during cooking, could have contributed to further reduce the accessibility of amylase to hydrolyze the starch (Zhang et al., 2015).

The active α-AI present in all bean biscuits (but the *lpa1*) represents an additional valuable feature, further contributing to the reduction of pGI (Table 5). Spadafranca et al. (2013) in a study performed on 12 volunteers to evaluate the effects of a standardized and purified *P. vulgaris* extract (PVE), containing 110.000 U of α-AI per 100 mg tablet, showed lower increments in glucose, insulin and C-peptide 3 h after meal consumption associated to 1 PVE tablet. We calculate that five B14 biscuits are equivalent to 1 PVE tablet, as they contain a comparable activity of α-AI.

On the basis of the above results, favorable implications for human health with a particular regard to the control of glycemia, especially in diabetic individuals and in the celiac population, can be hypothesized. For instance, for the celiac population, the enrichment of gluten-free baked products with fiber component is beneficial, since a general low intake of this food component has been reported (Lamacchia et al., 2014). In addition, although there is an on-going debate, the reduction of the dietary glycemic response following the consumption of lower GI food products can favorably influence several physiological parameters implicated as markers for conditions including overweight and obesity, diabetes mellitus and risk of coronary heart disease (Livesey et al., 2008; Brand-Miller et al., 2009). This aspect is also of particular interest concerning the celiac population, because several gluten-free foods exhibit higher GI than their gluten containing counterparts, due to the lack of the gluten matrix (Pellegrini and Agostoni, 2015).

In this work we considered two different gluten-free biscuit formulations, B22 and B29, and showed that they had lower pGI compared to control biscuit, confirming an additive contribution of bean flour. The acceptability of these products decreased with the increase of bean content, however the B22 gluten-free biscuits might represent a good compromise between health benefits (absence of gluten and lower pGI), expectations of celiac consumers and likeness. It should be pointed out that the consumers' test was a blind one and was not directed to celiac subjects. Appearance, flavor and texture are reported to be the most critical properties for consumer's acceptance of gluten-free products (Pagliarini et al., 2010). It is well known that gluten-free food products have a lower acceptability compared to those containing wheat (Pagliarini et al., 2010; Laureati et al., 2012). Moreover, it has been reported that differences in liking between celiac and non-celiac consumers may arise when judging gluten-free products (Laureati et al., 2012), with celiac consumers providing higher scores than non-celiac subjects to products of lower quality. In future studies, it is advisable to involve regular consumers of gluten-free products, since they are probably more sensitive to the differences in terms of liking to verify if a better score could be gained for B22 and B29 gluten-free biscuits.

In this context, future perspectives of this study should address the identification of the properties of appearance, smell, taste and texture that characterize bean-flour enriched biscuit formulations in order to define the sensory attributes that drive consumer's preference. Actually, this piece of research is intended as a pilot study carried out in order to give insights on the potentiality of Lady Joy bean flour as gluten-free ingredient, a fundamental topic, which truly needs to be investigated systematically.

## Conclusions

Food technology is important for the development of novel foods and food ingredients. Here we show the importance of the genetic and breeding approach to contribute and possibly facilitate the improvement of the primary product. In this work we show the feasibility of making nutritionally improved biscuits by the addition of bean flour of selected genotypes. We took advantage of a PHA inactive genotype (Lady Joy), which allows the direct use of flour without any thermal treatment. This resulted in biscuits containing fair amount of active α-AI, which contributes to lowering the pGI. We also proved that the use of the *lpa1* line could significantly contribute to the reduction of total phytates in the biscuits, a trait that is expected to increase minerals bioavailability. We are now working to combine the properties of cv. Lady Joy (absence of active PHA and very active α-AI) with the low phytate content of the *lpa1* mutant to produce a new common bean line that could be useful to address the health issue of diabetic individuals with micronutrient (iron, zinc, etc) deficiencies and that if combined with maize flours in the B22 formulation could meet the requirement of celiac disease affected patients, that are more exposed to the risk of developing type 1 diabetes. Finally these products could be further diversified and improved by using nutritionally enhanced maize flours from *lpa1* mutants, anthocyanins enriched lines and “high quality protein” maize lines (Azevedo and Arruda, 2010; Landoni et al., 2013; Holding, 2014; Petroni et al., 2014).

## Author contributions

FS conceptualized, designed, and supervised the project. MD, ML, RP, GG, PF carried out the experiments. FS, RB provided the bean genotypes and wrote the manuscript. FS, EC, ML, RP, EP, GG, RB regularly discussed the experiments, analyzed the results, provided useful suggestions during the project and critically revised the manuscript. All authors read and approved the final manuscript.

## Funding

This work was supported by the Programme FILAGRO “Strategie innovative e sostenibili per la filiera agroalimentare,” as part of the activities defined within the Accordo Quadro Consiglio Nazionale delle Ricerche and Regione Lombardia.

### Conflict of interest statement

The authors declare that the research was conducted in the absence of any commercial or financial relationships that could be construed as a potential conflict of interest.

## Appendix

**Table A1:** **Percent amino acid variations in biscuit samples containing different amounts of cv. Lady Joy bean flour compared to control biscuits**.

**Sample**	**Thr**	**Lys**	**Trp**	**Cys**	**Met**	**Met+Cys**	**Phe**	**Val**	**His**	**Ile**	**Asp**	**Glu**	**Pro**	**Gly**	**Ala**	**Leu**	**Tyr**	**Arg**	**Proteins**
B12	10.09	118.06	39.02	−2.86	20.57	7.59	30.87	26.01	−3.93	25.08	30.24	−1.53	0.21	19.40	15.19	12.05	17.33	84.14	14.86
B14	36.80	236.81	134.15	−5.71	22.70	6.96	55.67	43.75	41.92	57.59	41.43	16.74	35.85	49.16	21.01	21.33	26.00	83.82	37.49
B22	40.06	215.28	41.46	−10.86	34.04	9.18	51.72	9.68	54.15	47.06	78.32	−17.29	39.57	35.45	46.84	39.20	35.00	80.26	28.91
B24	40.36	240.97	95.12	−4.00	31.21	11.71	55.94	52.62	53.71	51.08	79.20	−4.38	52.13	46.49	39.49	30.06	33.67	93.85	36.11
B29	57.86	275.00	104.88	−6.29	37.59	13.29	73.09	45.97	61.57	64.40	99.48	−4.89	48.09	56.86	55.44	47.23	46.00	108.09	46.86
Average (B12–B29)	37.03	217.22	82.93	−5.94	29.22	9.75	53.46	35.60	41.48	49.04	65.73	−2.27	35.17	41.47	35.59	29.97	31.60	90.03	32.85
